# Hidradenocarcinoma of the Female Breast: A Surgical Approach to a Rare Skin Tumor

**DOI:** 10.7759/cureus.42577

**Published:** 2023-07-27

**Authors:** Duarte Gil Alves, Cláudia Araújo, Jessica Sousa, Alexandra Lapa, Joaquim Abreu de Sousa

**Affiliations:** 1 General Surgery, Hospital Dr. Nélio Mendonça, Funchal, PRT; 2 Surgical Oncology, Instituto Português de Oncologia do Porto, Porto, PRT; 3 Radiology, Centro Hospitalar Universitario do Porto EPE, Porto, PRT; 4 Anatomical Pathology, Instituto Português de Oncologia do Porto, Porto, PRT

**Keywords:** macroscopic human anatomy, surgical approach, malignant, partial mastectomy, wide local excision, acrospiroma, eccrine carcinoma, breast disease, malignant hidradenoma, hidradenocarcinoma

## Abstract

Clear-cell hidradenocarcinomas are extremely uncommon sweat gland tumors with a predilection for the head and neck. In the limited number of articles reporting breast involvement, the primary focus concerns this entity's histological and immunohistochemical characteristics.

Since hidradenocarcinomas of the breast have the potential to resemble a primary breast carcinoma closely, diagnosis may be challenging. Therefore, the authors report the first case of hidradenocarcinoma of the breast, which features its macroscopic morphology. In addition, to increase physicians’ awareness of this rare neoplasm, the article also aims to detail its surgical approach.

## Introduction

Hidradenocarcinomas are malignant adnexal tumors that develop from sweat glands, more specifically of eccrine or apocrine origin [1]. These neoplasms present a very low incidence, accounting for only 0.001% of all tumors [2]. Often affecting the head and neck, hidradenocarcinomas may rarely arise in the extremities, the trunk, or the abdomen [3]. The most affected age group falls within the range of 50 to 70 years old, with a slightly higher risk of incidence in females [4].

Although a fraction of hidradenocarcinomas can arise from the malignant transformation of pre-existing benign clear-cell hidradenomas, the majority occur de novo [5]. Commonly asymptomatic, hidradenocarcinomas of the breast appear as well demarcated firm nodular lesions, close to the nipple-areola complex, arising mainly from the dermis or less frequently from the breast parenchyma [6].

The disease course tends to be aggressive with a known local recurrence of 50%. Around 60% present with metastatic disease, mainly affecting regional lymph nodes [1]. Even though recently published studies have shed light on a more favorable prognosis for hidradenocarcinomas, the scarcity of known cases poses a notable obstacle in comprehending its behavior [7].

Considering there is no consensus guiding the proper management of hidradenocarcinomas owing to their rarity, wide local excision with at least a 3-cm surgical margin remains the cornerstone of treatment [2]. In some instances, an initial excisional biopsy or wide local excision can be performed, followed by a subsequent re-intervention to achieve a free margin resection and local control of the disease [8,9].

## Case presentation

A 92-year-old female with an Eastern Cooperative Oncology Group (ECOG) performance status of 3 and a medical history of hypertension, diabetes, and atrial fibrillation was referred to our clinic due to suspected breast cancer. The patient presented with a palpable, nontender mass lateral to the left breast’s areola, with skin retraction, which she initially noticed two years earlier.

**Figure 1:**
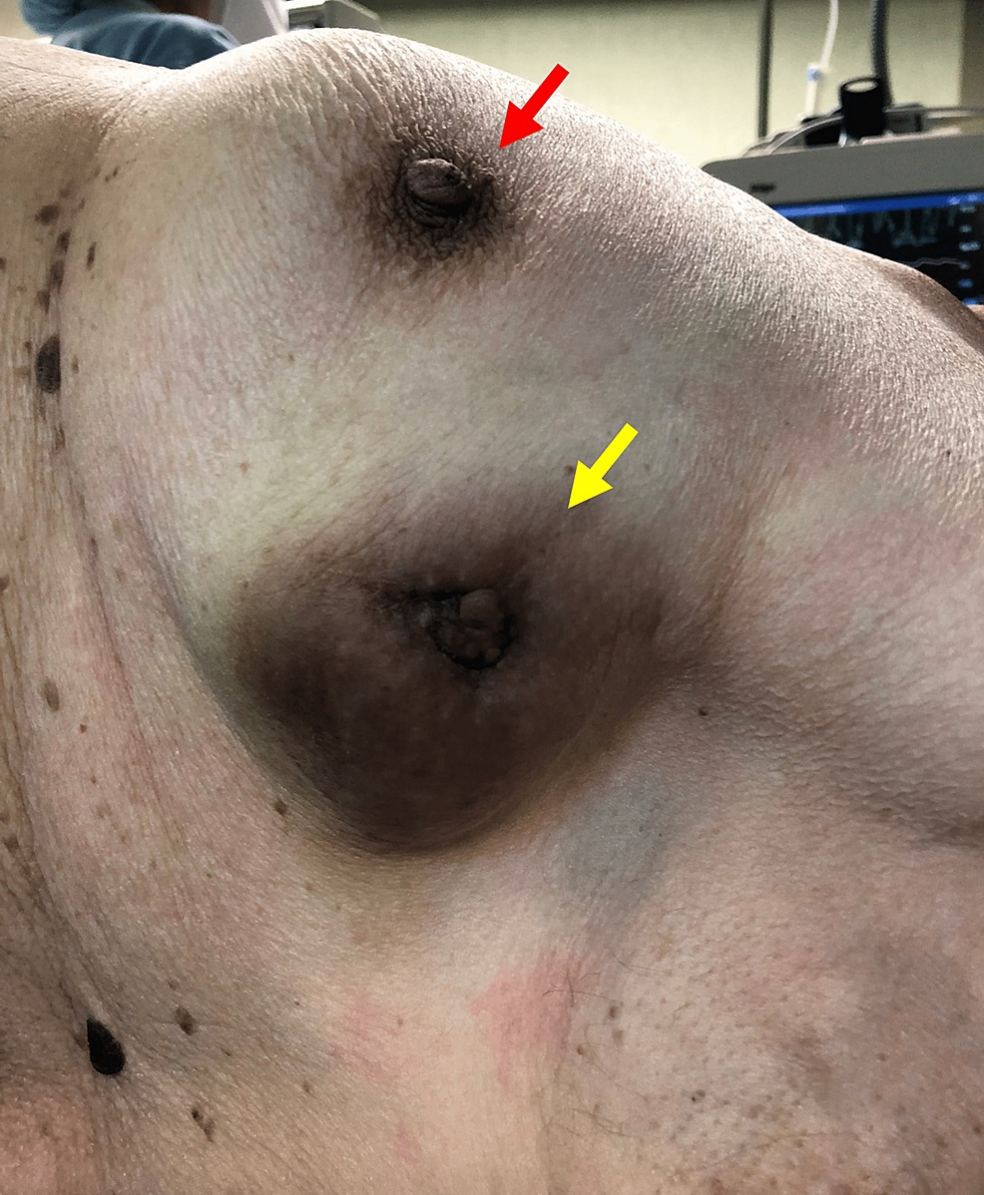
Patient placed in a dorsal decubitus position: left breast with the nipple-areola complex (red arrow) and the clear-cell hidradenocarcinoma in the upper outer quadrant (yellow arrow).

Physical examination revealed a superficial, mobile, firm, and well-circumscribed mass in the upper outer quadrant of the left breast. The ulcerated lesion, measuring a maximum diameter of 70 mm, resembled a second nipple-areola complex with a darkened color and a central protrusion (Figure 1). Ipsilateral axillary lymphadenopathy was palpable. A left breast ultrasound examination revealed a 50 mm × 70 mm solid lesion, with clear signs of skin invasion, in the upper outer quadrant. An 8 mm ipsilateral axillar lymphadenopathy was also identified. Ultrasound-guided fine needle aspiration of three cylinders of histological tissue from the breast mass was performed. The axillary lymphadenopathy also underwent fine needle aspiration.

Histopathological examination of the breast biopsy revealed a neoplasm with a solid pattern and nests of large cells delimited by sclera-hyaline bundles. The cells exhibited moderate pleomorphism, prominent nucleoli, and relatively abundant and eosinophilic cytoplasm. Foci of keratinization were also observed centrally, within these nests.

To determine the origin of the tumor (breast versus skin), immunocytochemical stains were performed. Positivity for p63, cytokeratins 5/6, and 7 (CK5/6, CK7) was observed, but GATA binding protein 3 (GATA-3) and estrogen and progesterone receptors (ER and PR) showed negativity. Therefore, based on the limited sample of the tumor obtained, a preliminary diagnosis of an adnexal tumor of the skin was reported, as characteristics of malignancy were not unequivocal.

The axillary lymph node biopsy was compatible with reactive lymphadenopathy, and no evidence of metastasis was identified.

**Figure 2:**
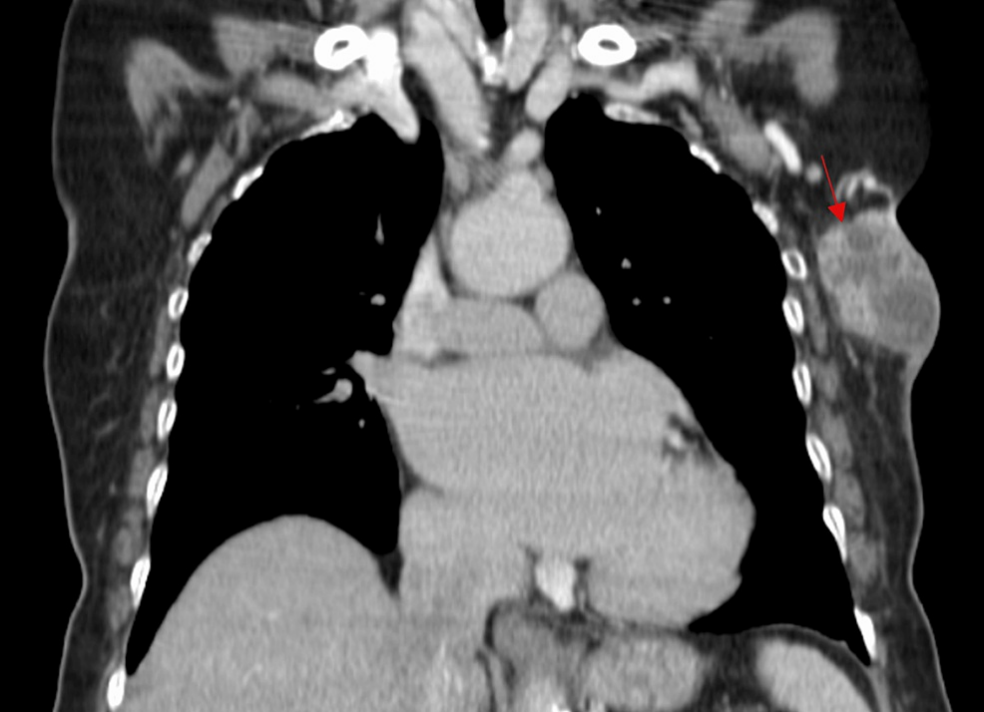
Coronal enhanced CT acquisition of the thorax revealing a lesion with heterogeneous contrast uptake, consisting of a solid and multicystic component, involving the upper outer quadrant of the left breast (red arrow). CT, computed tomography

**Figure 3:**
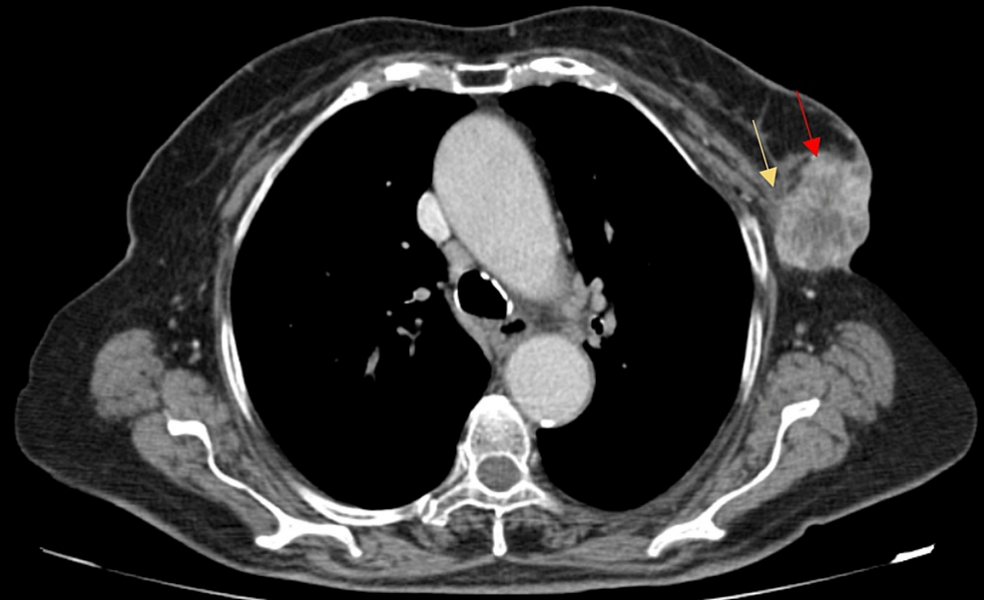
Axial enhanced CT acquisition of the thorax showing a lesion (red arrow) extending to the overlying skin and contacting deeply with the pectoral muscles (yellow arrow), yet without invading the endothoracic fascia. CT, computed tomography

A computed tomography (CT) scan of the thorax, abdomen, and pelvis with intravenous contrast revealed a lesion with heterogeneous contrast uptake, consisting of a solid and multicystic component, involving the upper outer quadrant of the left breast (Figure 2). The lesion, measuring 50 mm × 38 mm × 72 mm, showed extension to the overlying skin and deep contact with the pectoral muscles, yet without invading the endothoracic fascia (Figure 3). The presence of subcentimeter lymph nodes in the ipsilateral axilla level I was also noted. There was no evidence of metastatic disease.

**Figure 4:**
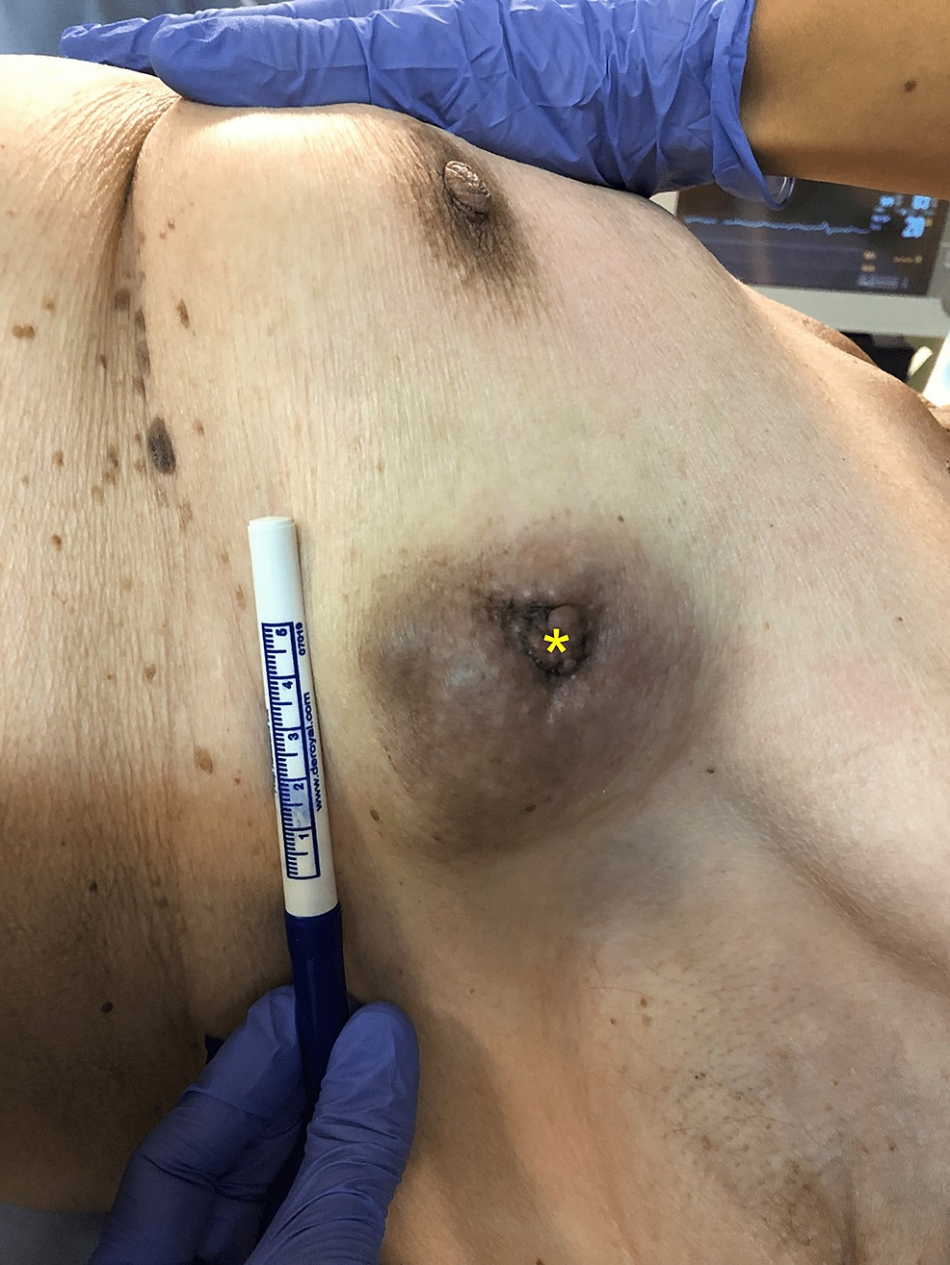
The ulcerated lesion, measuring a maximum diameter of 70 mm, with a darkened color and a central protrusion (yellow asterisk).

**Figure 5:**
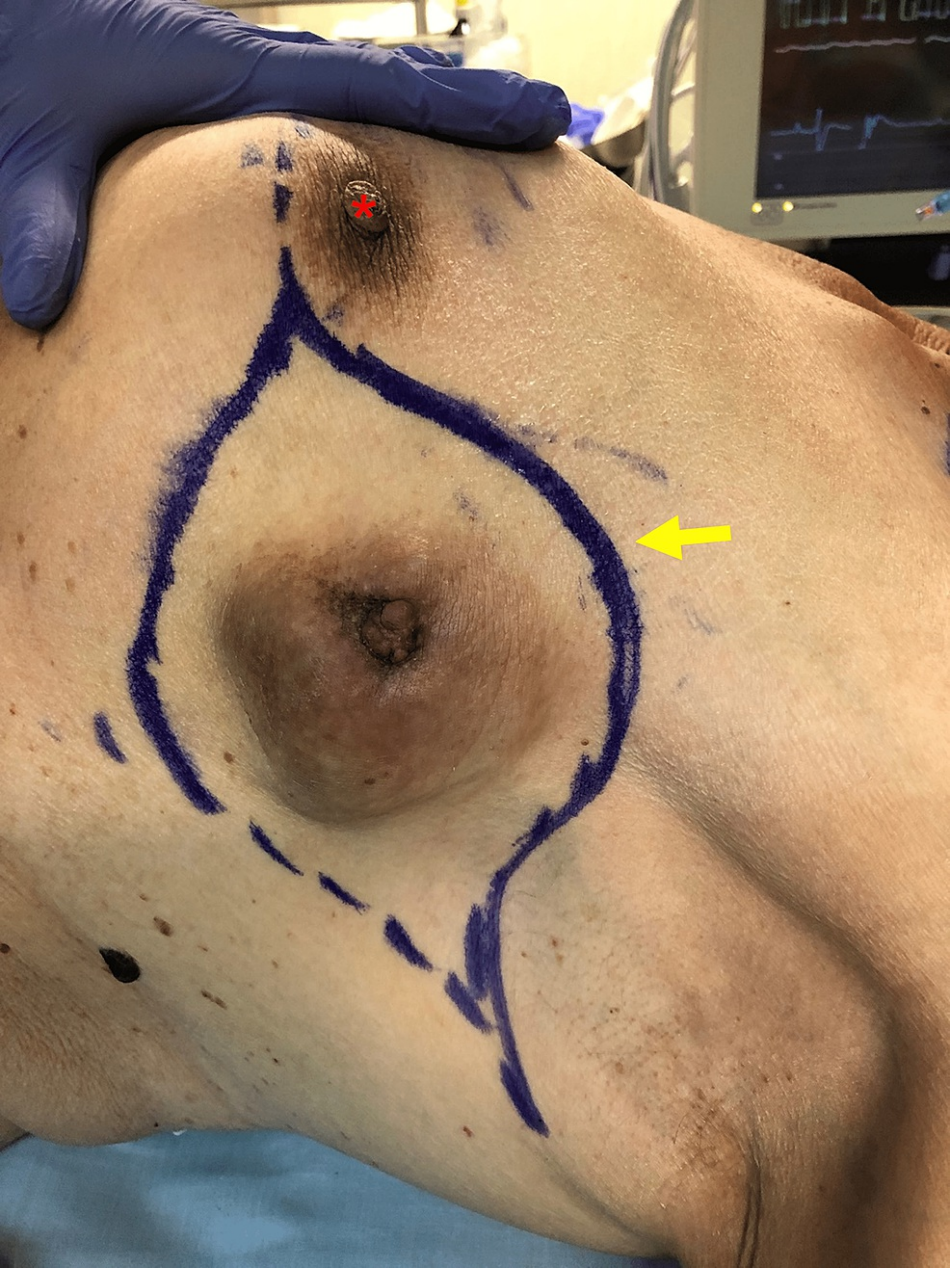
A lazy S-shaped incision with a 2-cm surgical margin was planned (yellow arrow), just lateral to the nipple-areola complex (red asterisk).

**Figure 6:**
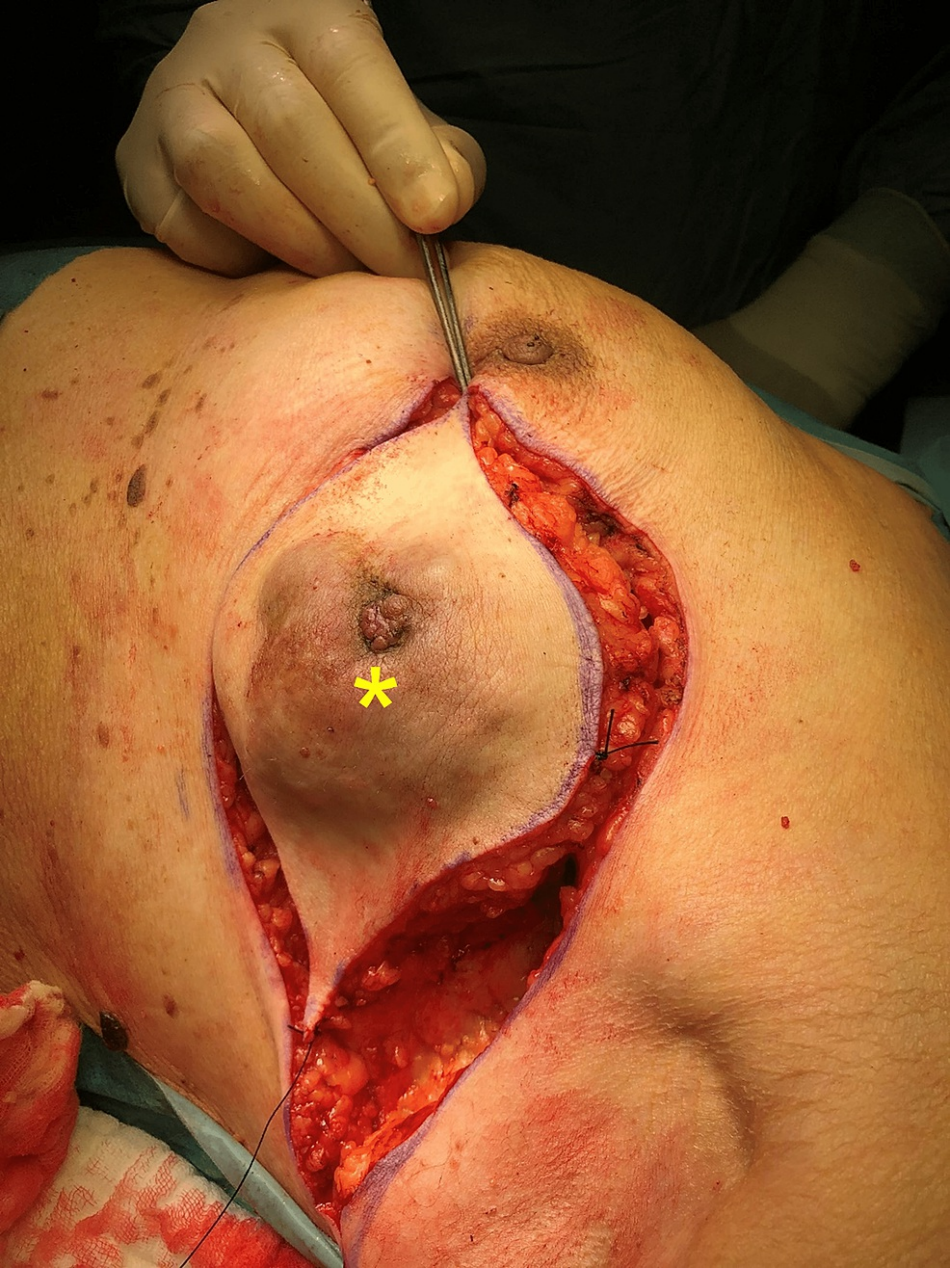
Surgical specimen in situ (yellow asterisk) after dissection proceeded deeply toward the major pectoral muscle.

**Figure 7:**
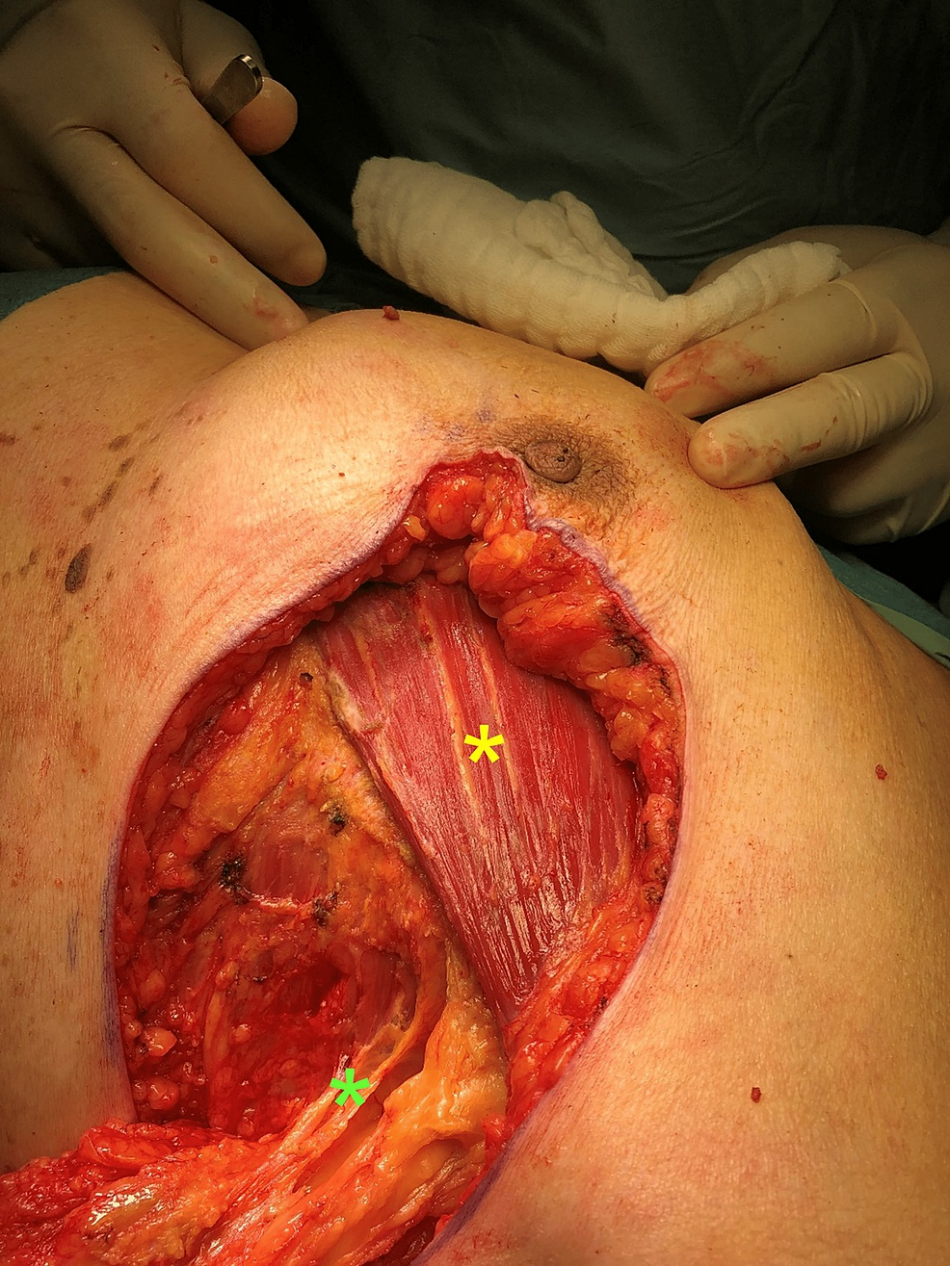
Final view of the surgical site before placement of the clips The major pectoral muscle is visible (yellow asterisk), as well as the level I axilla lymph nodes that were submitted to the en-bloc resection (green asterisk).

**Figure 8:**
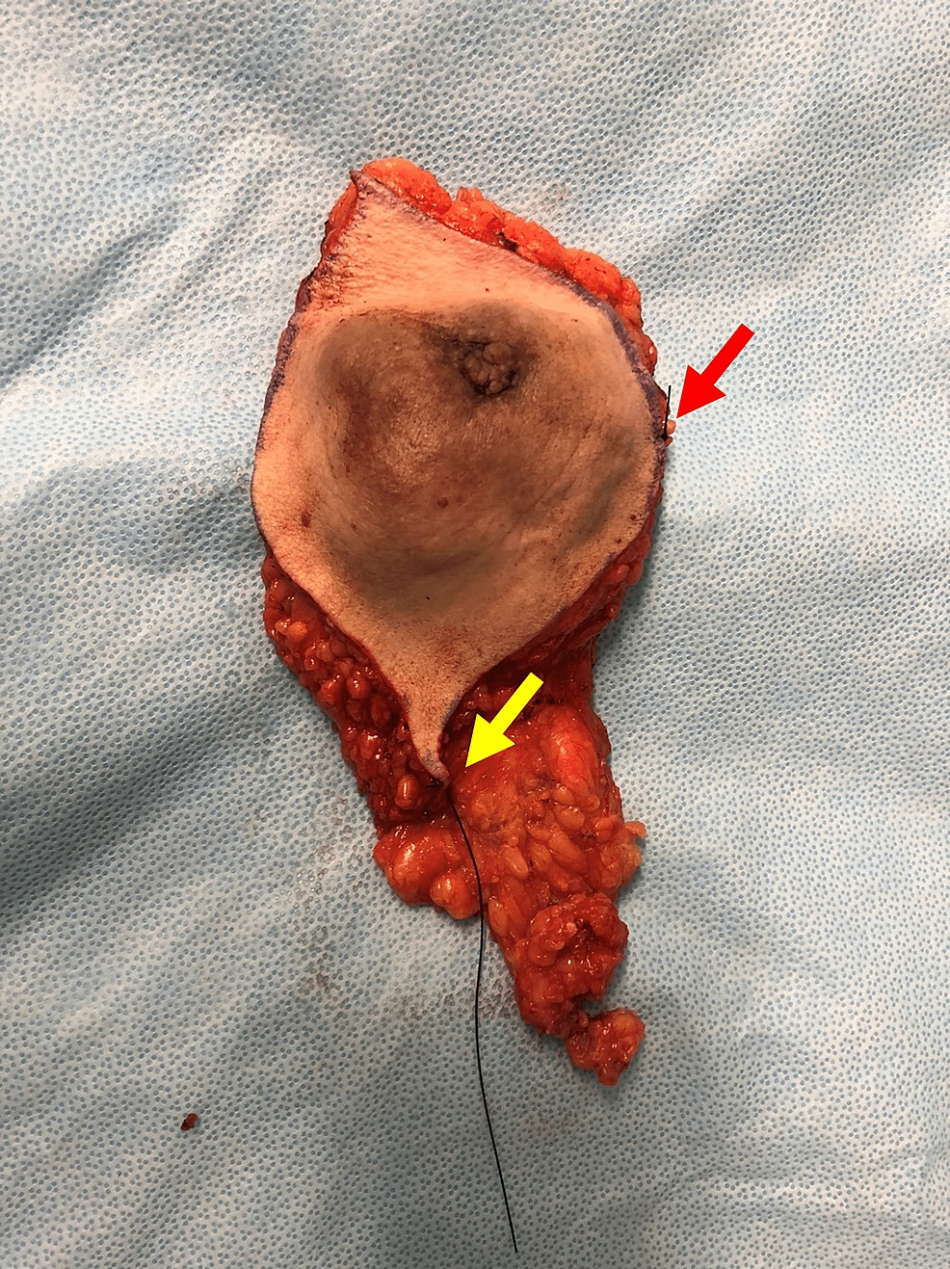
Surgical specimen with braided silk sutures marking the margins: a double suture on the superior margin (red arrow) and a long suture on the lateral margin (yellow arrow).

After a multidisciplinary team discussion, the patient was recommended a wide local excision consisting of a left partial mastectomy. Upon signing consent, she was put under general anesthesia and placed in a dorsal decubitus position (Figure 4). A lazy S-shaped incision with a 3 cm surgical margin was performed, allowing for a breast-sparing partial mastectomy (Figure 5). The dissection proceeded deeply toward the major pectoral muscle (Figure 6). En-bloc resection of lymph nodes from level I was performed (Figures 7-8). A continuous suction drain was placed in the surgical site. Clips were placed along all margins to mark the tumor bed, demarcating the extent of the dissection and guiding a subsequent re-intervention to extend margins, if needed. The subcutaneous fat was closed with a monofilament synthetic absorbable surgical suture, and a monofilament synthetic absorbable surgical suture was used for skin closure. On postoperative day four, as drainage volumes had gradually diminished, the surgical wound was reviewed, the suction drain was removed, and the patient was discharged.

**Figure 9:**
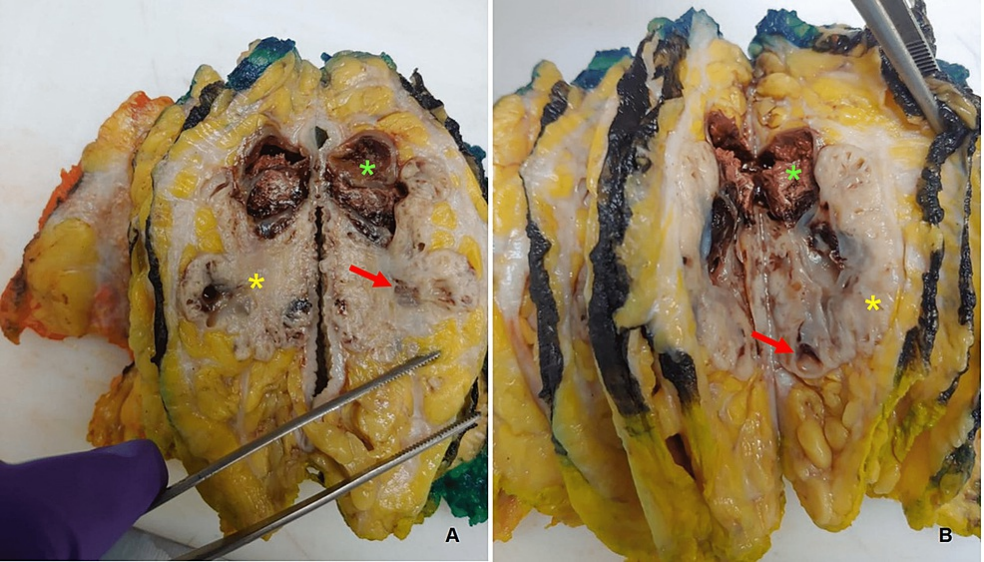
Surgical specimen after fixation. After section cutting (A and B), a solid (yellow asterisk) and cystic (red arrow) multilobulated mass with hemorrhagic and necrotic areas (green asterisk) can be observed.

**Figure 10:**
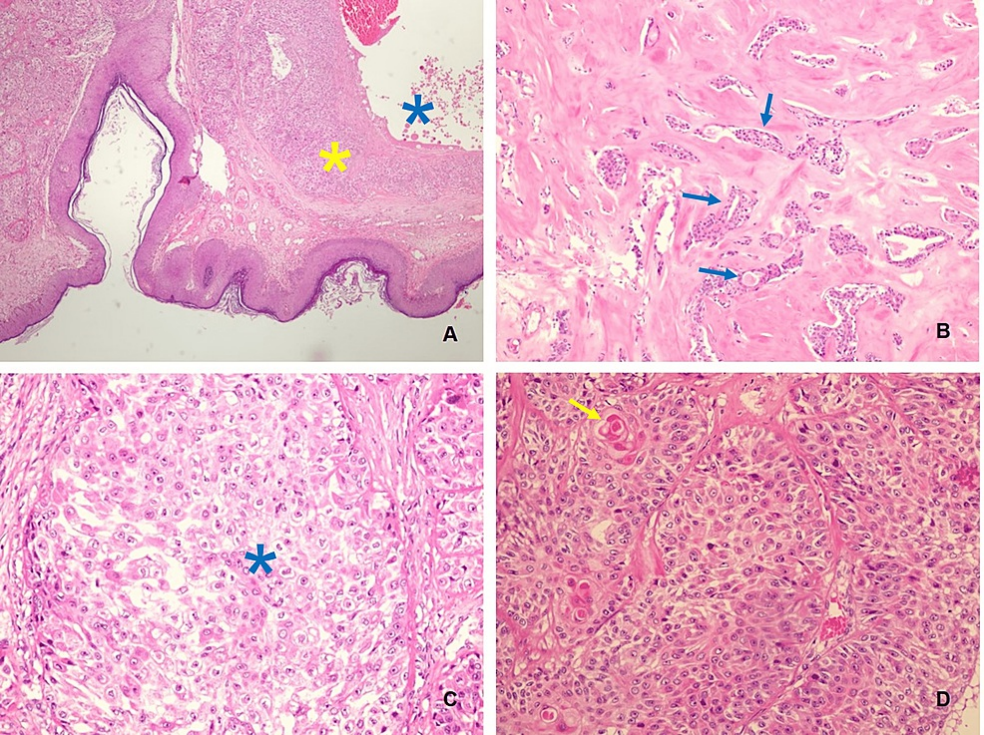
Examples of characteristic features observed in the histological examination. (A) Carcinoma with solid (yellow asterisk) and cystic (blue asterisk) components in the superficial dermis (HE  40×). (B) Tubular areas (blue arrows) in a dense stroma (HE 100×). (C) Clear-cell area (blue asterisk) (HE 200×). (D) Area with epidermoid/squamous differentiation, with the formation of keratin pearls (yellow arrow) (HE 200×). HE, hematoxylin and eosin

Histopathological examination of the surgical specimen revealed a 73 mm clear cell hidradenocarcinoma, centered in the dermis, with cells presenting similar characteristics to the ones previously reported in the biopsy, but also with areas of clear cells (Figures 9A-9B, 10A-10D). Necrosis, hemorrhage, and a mitotic index of 7 mitoses/1 mm² were observed. However, no perineural or vascular invasions were present. The surgical posterior and anterior margins were the closest surgical margins, with a 3 and 2 mm distance, respectively. The remaining margins were as follows: lateral 94 mm, medial 36 mm, superior 27 mm, and inferior 36 mm.

Moreover, an extensive immunocytochemical analysis was performed. The tumor cells exhibited positivity for p40, p63, and CK7. Additionally, they were positive for cytokeratin CAM 5.2, carcinoembryonic antigen (CEA), and epithelial membrane antigen (EMA), indicating ductal differentiation. P53 was positive, with wild-type immunophenotype and Ki-67 proliferation index staining reaching 40%. Stains for GATA-3, RE, RP, and human epidermal growth factor receptor 2 (HER-2) were negative, as well as stains for S100 protein, smooth muscle actin (SMA), and calponin, once again excluding the hypothesis of this tumor being a breast carcinoma.

Additionally, six lymph nodes were isolated from the specimen and were negative for metastasis.

Following the procedure, a subsequent multidisciplinary discussion took place to review the surgical outcomes and histological and immunohistochemical findings. The patient was advised to undergo a re-intervention to extend the margins, but she declined any further treatment. As of now, 20 months later, the patient is diligently attending regular annual follow-up visits and has shown no evidence of recurrence.

## Discussion

According to the literature, mainly due to its remarkably low incidence of 0.05%, less than 60 cases of hidradenocarcinoma have been reported [10]. Of those, only 11 articles concern instances of breast involvement [1,5,6,8,11-17]. Given that the diagnosis of hidradenocarcinoma relies on histological and immunohistochemical characteristics, these features have become the primary focus in the reported cases thus far. Nevertheless, to maintain a high index of suspicion during physical examination and to enable accurate differentiation between hidradenocarcinomas and primary breast carcinomas, physicians must be made aware of the macroscopic morphology of these neoplasms.

Immunohistochemical markers positivity for ER, PR, or HER2 may vary, yet most hidradenocarcinoma present positive staining for p40, p63, and CK5/6 [18]. Furthermore, Ki-67 proliferation index staining higher than 11% and a mitotic index over four mitoses per 10 high-power field (HPF) are features often present in the malignant variant of hidradenoma [19]. As of now, there have been no identified histologic characteristics that can serve as reliable indicators to predict hidradenocarcinoma aggressive behavior [6].

Ultrasound and magnetic resonance imaging can be used in local-regional staging, while CT and positron emission tomography scans are used to exclude distant metastatic disease. Additionally, CT and positron emission tomography scans may also be used to further evaluate a suspected recurrence during the follow-up period, allowing for early detection of local or distant disease [9].

A surgical approach aiming for a wide local excision with at least a 3 cm surgical margin is the most crucial key aspect regarding hidradenocarcinoma treatment [2]. To properly address these neoplasms, surgeons must be proficient in oncoplastic techniques to achieve a free-margin resection without compromising the local control of the disease or the aesthetic outcome [20]. In this case, since the patient refused a total mastectomy, surgeons chose to adapt the surgical technique aiming for minimal associated morbidity and excellent functional results.

Although radiation and chemotherapy have been previously utilized as adjuvant therapies, they have shown no significant impact on local disease control or overall survival, and as a result, they are not routinely recommended [1]. More recent treatment modalities, such as targeted therapy or electrochemotherapy, are considered valid therapeutic options, yet their efficacy requires further investigation [3].

## Conclusions

Hidradenocarcinoma is an extremely rare sweat gland tumor that a surgeon may perhaps encounter once in their lifetime. When these neoplasms are present in the breast, oncoplastic breast conservative surgery techniques must be adopted to optimize oncologic outcomes without compromising the aesthetic.

Since no adjuvant therapy has proven effective regarding local disease control or overall survival, the critical determinant of treatment success lies in the surgical approach and the achievement of a margin-free resection.
